# New records of a critically endangered shrew from Mexican cloud forests (Soricidae, *Cryptotis nelsoni*) and prospects for future field research

**DOI:** 10.3897/BDJ.6.e26667

**Published:** 2018-08-14

**Authors:** Lázaro Guevara, Víctor Sánchez-Cordero

**Affiliations:** 1 Departamento de Zoología, Instituto de Biología, Universidad Nacional Autónoma de México, Ciudad de Mexico, Mexico Departamento de Zoología, Instituto de Biología, Universidad Nacional Autónoma de México Ciudad de Mexico Mexico; 2 Departamento de Biología Evolutiva, Facultad de Ciencias, Universidad Nacional Autónoma de México, Ciudad de Mexico, Mexico Departamento de Biología Evolutiva, Facultad de Ciencias, Universidad Nacional Autónoma de México Ciudad de Mexico Mexico

**Keywords:** Endangered species, Eulipotyphla, Los Tuxtlas, niche modelling, sampling bias

## Abstract

The Nelson´s small-eared shrew, *Cryptotis nelsoni* (Merriam, 1895), is a critically endangered species, endemic to cloud forests in Los Tuxtlas, a mountain range along the Gulf of Mexico coast. This species is only known from the type locality and its surroundings. Here we present new records that extend its distribution approximately 7 km southeast of the type locality and report more specimens near to the type locality. We also identified climatically suitable areas for *C. nelsoni* using ecological niche modelling and investigated the sampling bias to identify poorly sampled areas in Los Tuxtlas. We suggest that the scarcity of records in other areas with suitable climatic conditions throughout Los Tuxtlas is a consequence of incomplete surveys. We strongly highlight the importance of continuing surveying this critically endangered shrew using more efficient sampling techniques to better understand its current distribution and conservation status. Despite all known localities occurring inside Los Tuxtlas Biosphere Reserve, deforestation and climate change still pose current and future threats to this species.

## Introduction

Monitoring populations of endangered species is a priority for assessing their conservation status and establish conservation actions (Guisan et al. 2006). However, many endangered species inhabit remote areas of limited access and finding populations to determine their distribution is often a difficult task. The Nelson’s small-eared shrew, *Cryptotis nelsoni* (Merriam, 1895), is a poorly known small mammal endemic to cloud forests in Los Tuxtlas, a mountain range along the Gulf of Mexico coast that is included within Los Tuxtlas Biosphere Reserve. Los Tuxtlas rises from sea level to the summit of three main volcanoes where access is difficult: San Martín Tuxtla volcano (1750 m), Santa Marta (1700 m) and San Martín Pajapan (1150 m).

*Cryptotis nelsoni* was discovered in 1894 by E.A. Goldman and E.W. Nelson (Merriam 1895, Goldman 1951) and was not seen again for more than a century, until it was rediscovered in 2003 (Cervantes and Guevara 2010). Since the known distribution of *C. nelsoni* is only a few locality points around the type locality in the San Martín Tuxtlas volcano and its habitat is rapidly changing or disappearing, this species is listed as “critically endangered” by the International Union for the Conservation of Nature and Natural Resources and “under special protection” by the Mexican government (Cervantes and Guevara 2010, Woodman et al. 2015). With so little known about this species, assessment of its conservation status has been based primarily on lack of knowledge.

A recent short-term field inventory of small mammals provided important new records for *C. nelsoni* in poorly explored areas around the San Martín Tuxtla volcano. Based on these new records and a search in natural history museums, our aim was to update information about the current distribution and conservation status of *C. nelsoni*. Additionally, we propose future field research guided by ecological niche modelling and taking into account the historical sampling effort in the region. We encourage continuing the monitoring of *C. nelsoni* to better understand its evolutionary history and conservation status.

## Materials and Methods

As part of a short-term biological inventory, we conducted our expedition in Los Tuxtlas between 14 and 16 September 2015, one of the rainiest months in the region. We used 100 unbaited pitfall traps in two main sites of cloud forest, spending only one night at each site. One site is located on the northeast face of the crater of the volcano San Martin Tuxtla, which is very close to the records reported by Cervantes and Guevara (2010). At this site, we divided traps into two different elevations (18°33'27"N, 95°11'24"W at 1,290 m; 18°33'54"N, 95°11'9"W at 1,460 m). The second site is located 7 km southeast of the crater, in a place known locally as Mastagaga hill (18°31'21"N, 95°8'31"W at 1,180 m). This hill is apparently connected with the volcano San Martín Tuxtla, but separated by a road crossing the cloud forest.

We followed standard recommendations on specimen capture, sacrifice and preparation (Sikes et al. 2011). The Mexican National Commission of Natural Protected Areas (CONANP) and the Mexican Ministry of the Environment (SEMARNAT) authorised the capture of small mammals in the Los Tuxtlas Biosphere Reserve, under the scientific collector permit issued to V. Sánchez-Cordero (permit FAUT–0006). We measured (mm) and weighed (g) all collected specimens in the field and deposited skins, skeletons, and/or tissues at the National Collection of Mammals (Colección Nacional de Mamíferos, CNMA), at the Institute of Biology, Universidad Nacional Autónoma de México, Mexico City, Mexico.

Taxonomic identification was based on qualitative morphological comparison of specimens recently collected against specimens previously identified as *Cryptotis nelsoni* (20 specimens), against a closely related species (*C. mexicanus*; 30 specimens) and against the geographically closer species (*C. parvus pueblensis*; 16 specimens). We recorded the following craniodental measurements for quantitative comparison: condylobasal length (CBL), breadth of palate across second molars (M2B), length of the upper molar toothrow (MTR) and height of coronoid process (HCP). All measurements were taken with a Mitutoyo electronic caliper at 0.1 mm precision under a stereomicroscope and are available as Suppl. Material (Suppl. material 1). To visualise the similarity in size and comparates of the previous qualitative identification, we generated scatterplots by comparing these quantitative variables.

We also visited the following natural history museums to investigate whether more records of *C. nelsoni* have been recently collected: National Collection of Mammals, Mexico City (CNMA); Collection of Mammals, El Colegio de la Frontera Sur, San Cristóbal de las Casas, Chiapas (ECO-SC-M); The University of Kansas Natural History Museum, Lawrence, Kansas (KU); Museum of Zoology ‘Alfonso L. Herrera’, Mexico City (MZFC); Collection of Mammals, Universidad Autónoma Metropolitana, Iztapalapa, Mexico City (UAMI); and National Museum of Natural History, Washington, District of Columbia (USNM).

To identify regions holding similar climatic conditions to where *Cryptotis nelsoni* is known to occur, we built an ecological niche model to estimate its potential distribution throughout Los Tuxtlas. We used a maximum entropy method in maxent 3.3.4, which is a presence-background technique that uses localities of known presence and a random sample of pixels from the study region to characterise the environments preferred by the species (Phillips et al. 2017). The relatively small number of localities of *C. nelsoni* makes it extremely challenging to characterise adequately its climatic requirements. To overcome this, we pooled together the localities of *C. nelsoni* with localities of their sister species, *C. mexicanus*, which is well-sampled and common through cloud forests from central-eastern Mexico (Guevara and Cervantes 2013). Pooling the occurrence records of closely related species into a single dataset increases the number of localities, improving the robustness of niche models for rare species (Qiao et al. 2017; occurrences records are available as Suppl. Material (Suppl. material 2). For predictor variable selection, model calibration and evaluation, we followed recent best-practices recommendations to estimate potential distributions of cloud forest species (Guevara et al. 2017). To display the final potential distribution, we divided the continuous prediction into a binary one (suitable vs. unsuitable) using the 10 percentile training presence threshold, where 10% of the localities having the lowest predicted values are excluded. Suitability values above this threshold were preserved in the original continuous format.

Finally, we tried to obtain an approximation of the sampling bias to identify well-surveyed areas in Los Tuxtlas. As a surrogate of sampling bias, we downloaded distributional records for small mammals collected in Los Tuxtlas from GBIF (https://doi.org/10.15468/dl.qpjjdk; accessed 21 April 2018) using a polygon as a filter (POLYGON(-95.5871, 17.86212; -94.3808, 17.86212; -94.3808, 18.71521; -95.5871, 18.71521; -95.5871, 17.86212). We only kept records from genera that can be collected with the same trapping techniques as shrews, using Museum special and Sherman traps (i.e. *Baiomys*, *Heteromys*, *Liomys*, *Microtus*, *Mus*, *Oligoryzomys*, *Peromyscus*, *Rattus*, *Reithrodontomys*, *Sciurus*, *Sigmodon*, *Thomomys*, *Tylomys* and *Xerospermophilus*). Finally, we included localities reported in a long-term inventory of small mammals in the region (González-Christen 2008). To obtain an approximation of range of elevations where the most sampling effort has been focused, we extracted the elevation of all the localities using a digital elevation map.

## Results

**Figure 1.**
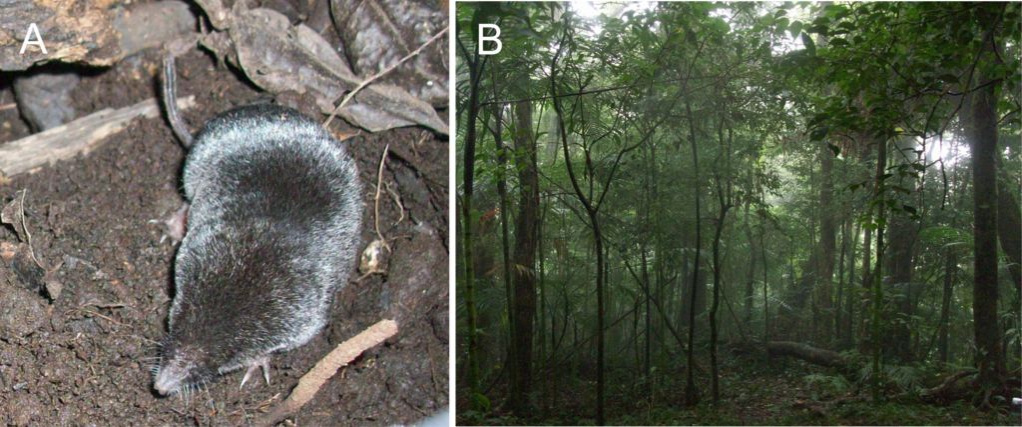
Specimen of the Nelson’s small-eared shrew (*Cryptotis nelsoni*) collected on 16 September 2015 in Mastagaga Hill, 7 km southeast the type locality (A). Typical cloud forest habitat of the highlands of the volcano San Martín Tuxtla (B).

**Figure 2.**
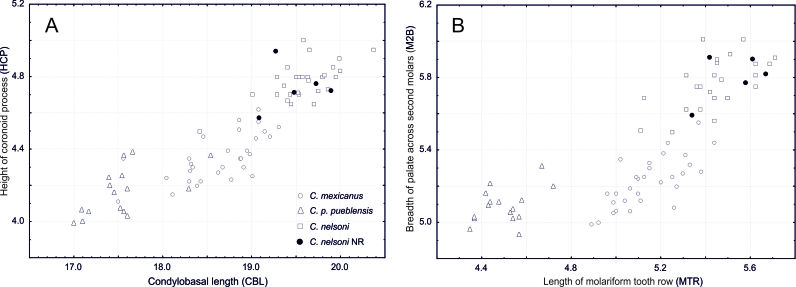
Scatterplots of quantitative variables for *Cryptotis nelsoni*, *C. mexicanus* and *C. parvus pueblensis* and specimens collected in the most recent exploration in Los Tuxtlas, a new record for *C. nelsoni* (NR). Condylobasal length versus height of coronoid process (A) and length of molariform tooth row versus breadth of palate across second molars (B) corroborate the identification of the new records as *C. nelsoni*.

Fieldwork resulted in 15 specimens of small-eared shrews (Fig. 1). At the first site, we collected four specimens at 1,460 m (CNMA 49294-49297) and five specimens at 1,290 m (CNMA 49289-49293); all of them were taken between montane cloud forest and elfin forests. At the second site (1,180 m), we found six specimens in small patches of secondary forest, surrounded by hiking trails (CNMA 49298-40303), which suggested some tolerance towards human disturbance of natural habitats. All the specimens displayed external characters of *Cryptotis nelsoni* reported previously (Cervantes and Guevara 2010, Choate 1970, Merriam 1895). They differed markedly from *C. mexicanus* and *C. p. pueblensis* in having a darker pelage and by their larger external body size, being similar to the specimens referred to *C. nelsoni*. The forefeet and fore claws were also distinctly broader and longer than *C. mexicanus* and *C. p. pueblensis*. The morphometric comparison corroborated our preliminary identification based on external characters. Scatterplots revealed that the collected specimens were larger than *C. mexicanus* and *C. p. pueblensis* in all variables examined and completely overlaps with the previously known specimens of *C. nelsoni* (Fig. 2).

We found 47 additional specimens collected in the vicinity of the type locality between November 2003 and March 2004, as reported by Cervantes and Guevara (2010). All specimens are deposited at the National Collection of Mammals at the Institute of Biology, UNAM. Detailed specimen information is provided as Suppl. Material (Suppl. material 3). To our knowledge, no other specimen of *C. nelsoni* has been collected in other parts of Los Tuxtlas.

**Figure 3.**
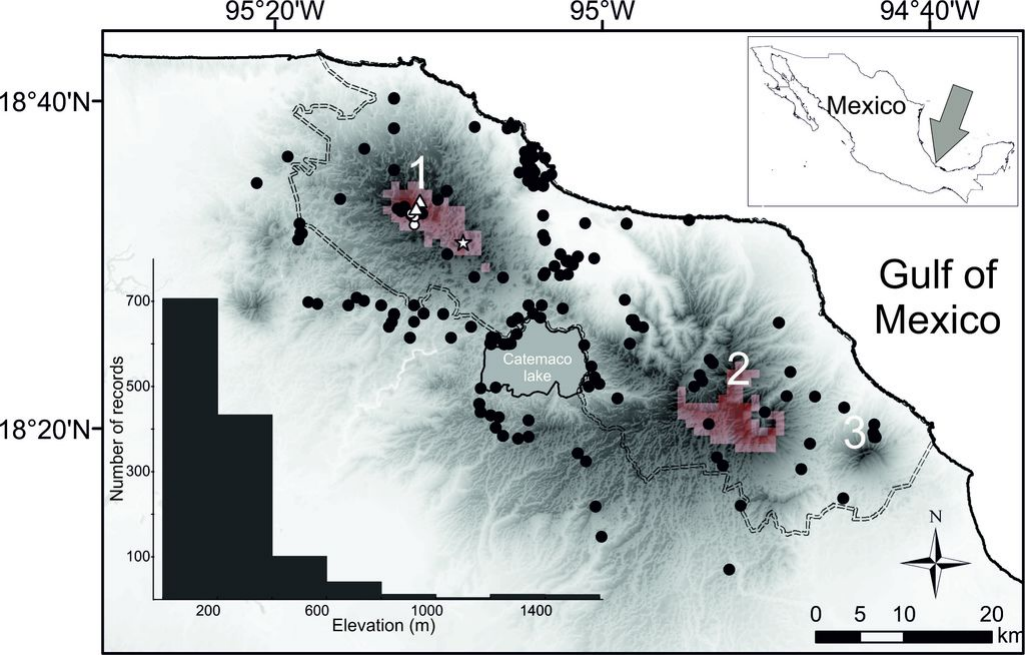
Map depicting known records of *Cryptotis nelsoni* in Los Tuxtlas, south-eastern Mexico. The three main volcanoes in Los Tuxtlas are indicated: (1) San Martin Tuxtla, (2) Santa Marta and (3) San Martín Pajapan. White circles are records from 2003 and 2004 in San Martín Tuxtla (Cervantes and Guevara 2010); white triangles and star (Mastagaga hill) are the new localities reported in our study. Black points are database records of other small mammals collected with the same sampling techniques as shrews, while the histogram shows the elevational distribution of these records. Darker colours indicate increasingly high elevation areas, while the dashed line depicts Los Tuxtlas Biosphere Reserve. Reddish pixels at 1 km^2^ indicate climatically suitable areas for *Cryptotis nelsoni* according to the maxent model; more reddish colours indicate more suitable habitat conditions.

The ecological niche model yielded a realistic estimation showing a prediction tightly associated with the distribution of cloud forests in Mexico. Suitable areas were identified only in San Martín Tuxtla and Santa Marta volcanoes, which were separated by unsuitable areas that suggest current geographic isolation between them. The highest suitability values largely corresponded to the highest and most inaccessible parts of Los Tuxtlas (see 1 and 2 inserted in Fig. 3).

Our search of surveys sampling bias at Los Tuxtlas led to 1,540 records from small mammals, after excluding data with missing locality, which resulted in 190 unique georeferenced localities. Although some areas of Los Tuxtlas have been relatively well-sampled, there are still remote areas lacking small mammal records probably due to insufficient or non-existent sampling effort (Fig. 3). These poorly sampled areas are mainly concentrated at high elevations, such as the main volcanoes in Los Tuxtlas (see histogram inserted in Fig. 3).

## Discussion

Our study highlights the importance of continued mammalian surveys in the Neotropics. In Los Tuxtlas, most of the small mammal sampling efforts have been conducted on lower and mid elevation range habitats, close to well-communicated towns and rural communities (Estrada et al. 1994, González-Christen 2008, Coates et al. 2017). Interestingly, there is a consistent absence of records of *Cryptotis nelsoni* in these lower and mid elevation sites, possibly reflecting true absences. This suggests that *C. nelsoni* is restricted to cloud forest habitats found in high elevations. Given that highlands of Sierra Santa Marta hold suitable climatic conditions, we propose that *C. nelsoni* can also occur in this isolated area. It is likely that the scarcity of records in this remote area is a consequence of incomplete surveys.

We believe that our sampling protocol using pitfall traps increased the efficiency of our inventory, as has been suggested for collecting shrews in the Neotropics (Guevara 2017, Umetsu et al. 2006, Woodman et al. 2012). Then, by using ecological niche modelling, combined with proper surveying techniques, the efficiency of sampling populations of critically endangered small mammals can be increased. Determining the current distribution of *C. nelsoni* is a first step to provide crucial information for understanding its evolutionary history and conservation status, as well as establishing long-term conservation actions in Los Tuxtlas. We urge the initiation of long-term surveys in poorly sampled and climatically suitable areas to accurately characterise the geographic distribution of this endangered species. Fortunately, all known and potential localities of *C. nelsoni* are included within Los Tuxtlas Biosphere Reserve, highlighting its importance in maintaining a high species richness and endemism. Nonetheless, regional deforestation and climate change still pose conservation threats to this shrew (Dirzo and García 1992, Rojas-Soto et al. 2012).

## Supplementary Material

Supplementary material 1 Cranio-mandibular measurements used for the quantitative comparison Data type: Morphological information
Brief description: Cranio-mandibular measurements used for the quantitative comparison and taxonomic identification
File: oo_202812.xls Lázaro Guevara, Víctor Sánchez-Cordero

Supplementary material 2 Occurrences records used to calibrate the ecological niche model Data type: Occurrences
Brief description: Occurrences records used to calibrate niche models. It includes records for Cryptotis mexicanus and C. nelsoni pooled together
File: oo_214624.csv Lázaro Guevara, Víctor Sánchez-Cordero

Supplementary material 3 Occurrence data of the Nelson´s small-eared shrew, *Cryptotis nelsoni* Data type: Occurrences
Brief description: Voucher information of Cryptotis nelsoni housed in natural history museums
File: oo_214625.xls Lázaro Guevara, Víctor Sánchez-Cordero
